# Yokukansan Improves Mechanical Allodynia through the Regulation of Interleukin-6 Expression in the Spinal Cord in Mice with Neuropathic Pain

**DOI:** 10.1155/2015/870687

**Published:** 2015-03-18

**Authors:** Shigeru Ebisawa, Tsugunobu Andoh, Yutaka Shimada, Yasushi Kuraishi

**Affiliations:** ^1^Department of Japanese Oriental Medicine, Graduate School of Medicine and Pharmaceutical Sciences, University of Toyama, Toyama 930-0194, Japan; ^2^Department of Applied Pharmacology, Graduate School of Medicine and Pharmaceutical Sciences, University of Toyama, Toyama 930-0194, Japan

## Abstract

Neuropathic pain is caused by nerve injury. Yokukansan (Yi-Gan San), a traditional Japanese (Kampo) medicine, has been widely used for neuropathic pain control. However, the analgesic mechanisms remain unknown. In this study, we investigated the analgesic mechanisms of yokukansan in a mouse model of neuropathic pain. Partial sciatic nerve ligation (PSL) induced mechanical allodynia in mice. Repetitive oral administration of the extracts of yokukansan and the constituent herbal medicine Atractylodis Lanceae Rhizoma, but not Glycyrrhizae Radix, relieved mechanical allodynia in the PSL mice and inhibited the PSL-induced expression of interleukin- (IL-) 6 mRNA in the spinal cord. Yokukansan did not attenuate intrathecal IL-6-induced mechanical allodynia. IL-6 immunoreactivity was detected in microglia and astrocytes in the spinal dorsal horn. These results suggest that yokukansan relieves mechanical allodynia in PSL mice by regulating the expression of IL-6 in astrocytes and/or microglia in the spinal cord. In addition, the components of Atractylodis Lanceae Rhizoma, one of the constituent herbal medicines in yokukansan, may play an important role in the regulation of IL-6 expression and neuropathic pain control.

## 1. Introduction

Neuropathic pain is primarily induced by sensory nerve damage and is very difficult to control. Several animal models of neuropathic pain have been developed [1] and evaluated, with the findings indicating that ATP, cytokines (e.g., interleukin- (IL-) 6, IL-1*β*, and tumor necrosis factor alpha), nitric oxide, prostaglandins, and brain-derived neurotrophic factor released from microglia and astrocytes in the spinal cord contribute to neuropathic pain [2]. However, few drugs are available for the regulation of these factors to control neuropathic pain.

Yokukansan (Yi-Gan San) is a traditional Japanese (Kampo) formula comprising seven herbal medicines (Atractylodis Lanceae Rhizoma, Hoelen, Cnidii Rhizoma, Uncariae Uncis Cum Ramulus, Angelicae Radix, Bupleuri Radix, and Glycyrrhizae Radix) in a specific ratio as shown in Table 1. Yokukansan is used to control nighttime crying in children and to treat insomnia and neurosis. Recent reports also showed that yokukansan controls hallucinations and aggravation of dementia-associated symptoms and neuropathic pain [3, 4]. However, the inhibitory mechanisms of yokukansan for the control of neuropathic pain remain unclear. Therefore, in the present study, we investigated the therapeutic effects of yokukansan on neuropathic pain and the underlying mechanisms in a mouse model of neuropathic pain induced by partial sciatic nerve ligation (PSL).

IL-6 is well known as a proinflammatory cytokine. In animal model of neuropathic pain induced by PSL, the expression of spinal IL-6 increases [5, 6] and an intrathecal treatment with recombinant mouse gp130/Fc chimera protein, which is a specific inhibitor of endogenous IL-6 [7–9], inhibits the mechanical allodynia [10], suggesting that spinal IL-6 plays an important role in the development of neuropathic pain. Thus, we also investigated whether the regulation of IL-6 was involved in the action of yokukansan.

## 2. Materials and Methods

### 2.1. Animals

Five-week-old male ICR mice (Japan SLC, Shizuoka) were used in this study. Four to six mice per cage were housed in a room with controlled temperature (22 ± 1°C) and light (lights on from 7:00 AM to 7:00 PM). Food and water were available ad libitum. This study was performed after obtaining approval from the Animal Care Committee of the University of Toyama. The pain test was performed according to the guidelines on ethical standards for investigations of experimental pain in animals [11].

### 2.2. Drugs

Dried extracts of yokukansan (Lot 211054010, TJ-54), Atractylodis Lanceae Rhizoma (Lot 2131005010), and Glycyrrhizae Radix (Lot 2131013010) were obtained from Tsumura & Co. (Tokyo). These were dissolved in 5% gum arabic and administered by oral gavage. The drug administration schedule is shown in Figure 2(a). IL-6 was purchased from PeproTech Inc. (Rocky Hill, NJ, USA) and dissolved in phosphate-buffered saline (PBS).

### 2.3. Surgical Procedure

Mice were anesthetized with intraperitoneal sodium pentobarbital (70 mg/kg, Sigma-Aldrich, St. Louis, MO, USA). Partial nerve injury models were prepared by applying a tight 9-0 silk suture ligature at the anterior part around approximately 1/3 to 1/2 the diameter of the right sciatic nerve (PSL), as described previously [12, 13]. The sham group underwent all procedures except ligation.

### 2.4. Intrathecal Injection

Intrathecal (i.t.) injection of IL-6 or the vehicle (PBS) was performed as described by Hylden and Wilcox [14] using a 25 *μ*L Hamilton syringe (Hamilton Company, NV, USA) with 30-gauge needle. The needle was inserted into the intervertebral space of the spinal cord of unanesthetized mice. A reflexive flick of the tail was considered to indicate the accuracy of each injection. The volume for intrathecal injection was 5 *μ*L.

### 2.5. Behavioral Experiments

Mechanical allodynia in the hind paw was evaluated using von Frey filaments (North Coast Medical Inc., Morgan Hill, CA, USA). The mice were individually placed in a plastic cage with a metal mesh bottom. The animals were allowed to adapt for approximately 1 h or until exploratory behavior ceased. A von Frey filament with a bending force of 0.16 g was perpendicularly pressed against the lateral edge of the planter hind paw surface until it bowed slightly. The responses to these stimuli were ranked as follows: 0: no response; 1: lifting of the hind paw; and 2: immediate flinching or licking of the hind paw. The stimulus was applied six times to the hind paw at intervals of several seconds, and the average of the six values served as the pain-related score, as described previously [15]. Mice with scores of ≥1 before surgery or <1 after PSL were excluded.

### 2.6. Isolation of the Spinal Cord

Mice were anesthetized with intraperitoneal sodium pentobarbital (70 mg/kg, Sigma-Aldrich) and transcardially perfused with phosphate-buffered saline (PBS). The L3–L6 level of the spinal cord was immediately removed.

### 2.7. Real Time Reverse Transcription Polymerase Chain Reaction (PCR)

The total RNA in the L3–L6 level of the spinal cord of the ipsilateral posterior side was extracted using the RNeasy Lipid Tissue Mini Kit (Qiagen, Heiden, Germany) according to the manufacturer's instructions. Quantitative PCR was performed using the One-Step SYBR PrimeScript RT PCR Kit II (TAKARA Bio Inc., Shiga) using the Thermal Cycler Dice Real Time System (TAKARA), and data were analyzed using the Thermal Cycler Dice Real Time System Software Ver. 4.02B (TAKARA). The sequences of the primer pairs were as follows: IL-6: TTTCCTCTGGTCTTCTGGAGTA (forward) and CTCTGAAGGACTCTGGCTTTG (reverse) and GAPDH: TCAACGGCACAGTCAAGG (forward) and ACTCCACGACATACTCAG (reverse).

### 2.8. Immunohistochemistry

The lumbar spinal cord sections were fixed in 4% paraformaldehyde for 2 h at room temperature, permeated with 30% (w/v) sucrose solution in PBS for 72 h at 4°C, and frozen in an embedding compound (Sakura Finetechnical, Tokyo) on dry ice. The frozen samples were then sectioned at 16 *μ*m, and the sections were thaw-mounted on glass slides and incubated in blocking solution (1.5% fetal bovine serum) for 30 min at room temperature, followed by incubation for 24 h at 4°C with primary antibodies for glial fibrillary acidic protein (GFAP; rat, 1 : 20000, Invitrogen, Inc., Carlsbad, CA, USA) as a marker for astrocytic activation, ionized calcium-binding adaptor molecule 1 (Iba-1; rabbit, 1 : 2000, Wako, Osaka) as a marker for microglia activation, NeuN (mouse, 1 : 100, EMD Millipore, MA, USA) as a marker for neurons, and IL-6 (goat, 1 : 1000, R&D systems, Minneapolis, MN, USA). The antibody was then rinsed and incubated with an appropriate secondary antibody conjugated with Alexa 488 and Alexa 594 (Molecular probes, Inc., Eugene, OR, USA) or Cy3 (anti-rat IgG, Rockland Immunochemicals, Inc., Gilbertsville, PA, USA) for 2 h at room temperature. Fluorescence by immunolabeling was detected using a laser scanning microscope (LSM780, Carl Zeiss, Oberkochen, Germany).

### 2.9. Data Analysis

Data are expressed as means and standard errors of the means. Statistical analyses were performed using two-way repeated measures analysis of variance (ANOVA) with a post hoc Holm-Sidak test or Student's *t*-test using SigmaPlot graphing and statistical software (version 11; Systat Software, Inc., Chicago, IL, USA). Differences were considered statistically significant at *P* < 0.05.

## 3. Results

### 3.1. Effects of Yokukansan and the Constituent Herbal Medicines on Mechanical Allodynia in PSL Mice

**Figure 1:**
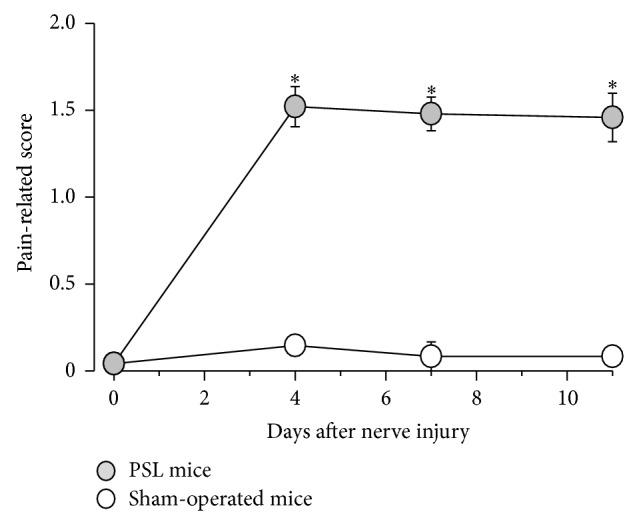
Mechanical allodynia in mice with partial sciatic nerve ligation (PSL). PSL or a sham operation was performed on day 0. Mechanical allodynia was tested by punctate stimulation using a von Frey filament with a bending force of 0.16 g applied to the footpad of the paw on the PSL side. Data are presented as means ± standard errors of the means (*n* = 8). ^*^ *P* < 0.001 compared with the sham mice (Holm-Sidak test).

**Figure 2:**
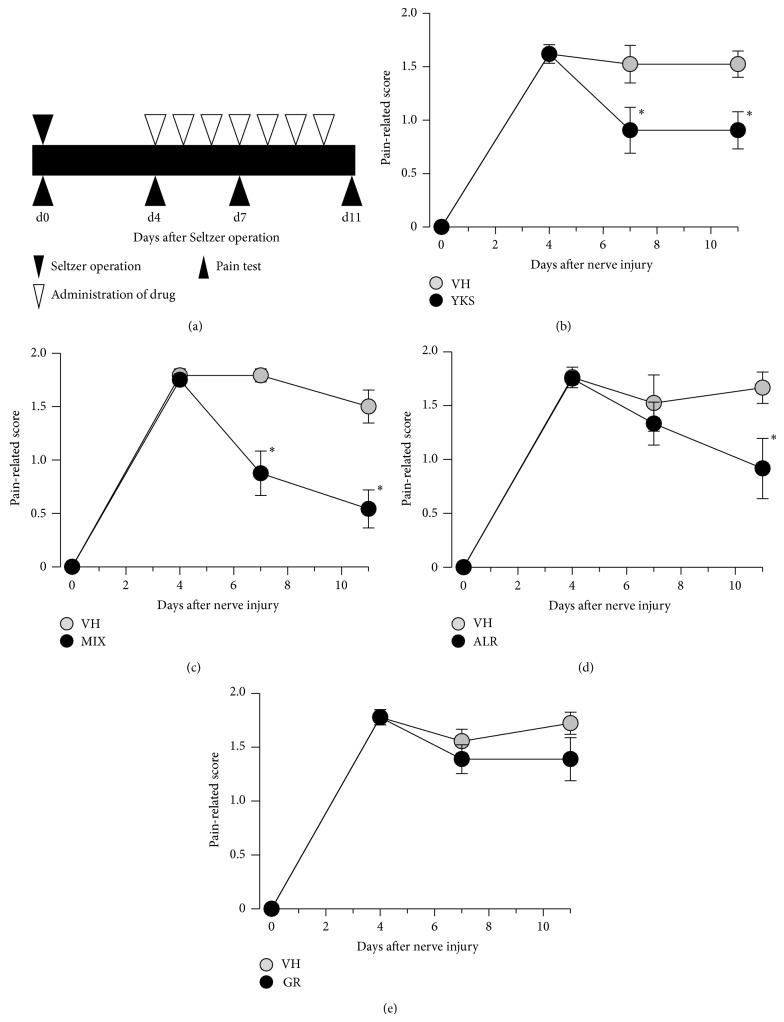
Effects of yokukansan and the constituent herbal medicines on mechanical allodynia in mice with partial sciatic nerve ligation (PSL). (a) Time schedule of drug administration and the evaluation of mechanical allodynia. Partial sciatic nerve ligation (PSL) was performed on day 0. Mechanical allodynia was tested by punctate stimulation using a von Frey filament with a bending force of 0.16 g applied to the footpad of the paw on the PSL side. Yokukansan extract (YKS, 1 g/kg) (b), a mixture (MIX) of Glycyrrhizae Radix (GR, 100 mg/kg) and Atractylodis Lanceae Rhizoma (ALR, 200 mg/kg) extracts (c), ALR extract (ALR, 200 mg/kg) (d), GR extract (100 mg/kg) (e), or vehicle (VH) was orally administered once a day from day 4 after surgery for 7 days. Data are presented as means ± standard errors of the means (*n* = 7-8). ^*^ *P* < 0.001, compared with the vehicle-treated group (Holm-Sidak test).

Mechanical allodynia in PSL mice, but not sham mice, was evident on day 4 after surgery and continued until at least day 11 (Figure 1: main effect of surgery, *F*_1,14_ = 207.646, *P* < 0.001; interaction between surgery and time, *F*_3,42_ = 37.312, *P* < 0.001 (two-way repeated measures ANOVA)). Compared with that in the vehicle-treated mice, mechanical allodynia in the PSL mice was significantly attenuated by the repetitive oral administration of 1 g/kg yokukansan extract [16–18] (Figure 2(b): main effect of surgery, *F*_1,12_ = 5.947, *P* < 0.05; interaction between surgery and time, *F*_2,24_ = 4.949, *P* < 0.05 (two-way repeated measures ANOVA)). Our preliminary experiment showed that keishikajutsubuto attenuates mechanical allodynia in PSL mice (data not shown). Keishikajutsubuto comprises Cinamomi Cortex, Paeoniae Radix, Atractylodis Lanceae Rhizoma, Jujubae Fructus, Glycyrrhizae Radix, Zingiberis Rhizoma, and Aconiti Tuber. Therefore, we investigated the antiallodynic effects of the extracts of Glycyrrhizae Radix and Atractylodis Lanceae Rhizoma, which are components of both yokukansan and keishikajutsubuto. The dose of Atractylodis Lanceae Rhizoma and Glycyrrhizae Radix was determined in reference to the ratio derived by preextraction of these Kampo components. Compared with that in the vehicle-treated mice, mechanical allodynia in the PSL mice was significantly attenuated by the repetitive oral administration of a mixture of Glycyrrhizae Radix (100 mg/kg) and Atractylodis Lanceae Rhizoma (200 mg/kg) extracts (Figure 2(c): main effect of surgery, *F*_1,14_ = 21.160, *P* < 0.001; interaction between surgery and time, *F*_2,28_ = 10.455, *P* < 0.001 (two-way repeated measures ANOVA)) and Atractylodis Lanceae Rhizoma extract alone (200 mg/kg; Figure 2(d): main effect of surgery, *F*_1,13_ = 5.576, *P* < 0.05; interaction between surgery and time, *F*_2,26_ = 4.28, *P* < 0.05 (two-way repeated measures ANOVA)) but not by administration of Glycyrrhizae Radix extract alone (100 mg/kg; Figure 2(e)).

### 3.2. Effects of Yokukansan on Mechanical Allodynia Induced by Intrathecal Injection of IL-6 in Naïve Mice

**Figure 3:**
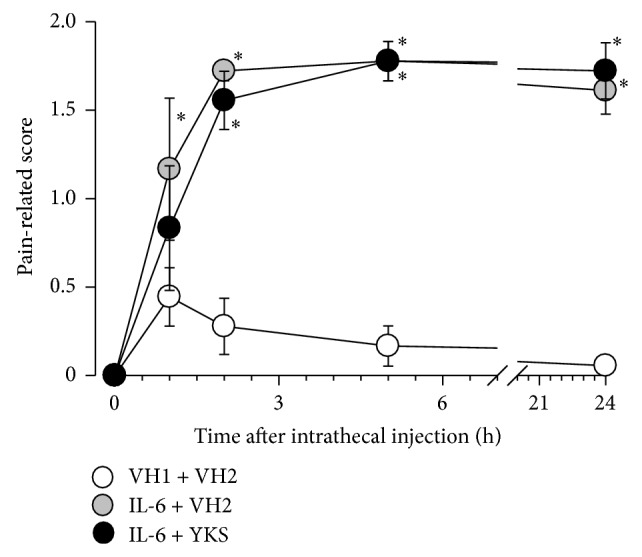
Effect of yokukansan on mechanical allodynia induced by intrathecal injection of interleukin- (IL-) 6 in naïve mice. Mechanical allodynia was tested by punctate stimulation using a von Frey filament with a bending force of 0.16 g applied to the footpad of the paw on right side. Yokukansan extract (YKS, 1 g/kg) or vehicle (VH2: tap water) was orally administered 2 h before intrathecal injection of IL-6 or PBS (VH1). Data are presented as means ± standard errors of the means (*n* = 6).

Imai et al. [10] showed that spinal IL-6 contributes to the induction of neuropathic pain in PSL mice. Thus, in this experiment, we investigated whether yokukansan inhibited the intrathecal IL-6-induced mechanical allodynia. An intrathecal injection of IL-6 (1 pmol/site) [10] elicited mechanical allodynia within 2 h after the injection (Figure 3). The oral administration of yokukansan (1 g/kg) 2 h before IL-6 injection did not affect the mechanical allodynia (Figure 3).

### 3.3. Effects of Yokukansan and the Constituent Herbal Medicines on the Expression of IL-6 mRNA in the Spinal Cord of PSL Mice

**Figure 4:**
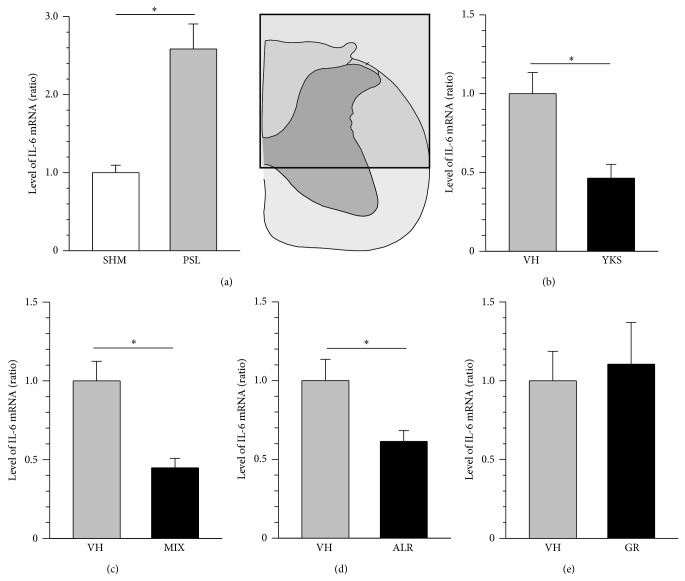
Effects of yokukansan and the constituent herbal medicines on the expression of interleukin- (IL-) 6 mRNA in the spinal cord of mice with mechanical allodynia induced by partial sciatic nerve ligation (PSL). The total RNA of the L3–L6 level of the spinal cord of the ipsilateral posterior side was extracted 11 days after surgery. Expression of IL-6 mRNA in the spinal cord was determined using real time polymerase chain reaction. (a) The expression of IL-6 mRNA in the spinal cord of the sham (SHM) and PSL-treated mice. Yokukansan extract (YKS, 1 g/kg) (b), a mixture (MIX) of Glycyrrhizae Radix (GR, 100 mg/kg) and Atractylodis Lanceae Rhizoma (ALR, 200 mg/kg) extracts (c), ALR extract (200 mg/kg) (d), GR extract (100 mg/kg) (e), or vehicle (VH) was orally administered once a day from day 4 after surgery for 7 days. Expression of IL-6 mRNA in each sample was normalized to that of glyceraldehyde-3-phosphate dehydrogenase, followed by normalization to its expression level in the controls. The upper right panel shows the site of evaluation of IL-6 mRNA expression. Data are presented as means ± standard errors of the means (*n* = 4–8). ^*^ *P* < 0.001, compared with the sham mice or VH-treated mice (Student's *t*-test).

In this experiment, we investigated the expression of IL-6 mRNA using the L3–L6 level of the spinal cord of the ipsilateral posterior side in PSL mice treated with traditional herbal medicines for 7 days. The expression of IL-6 mRNA in the spinal cord was significantly increased in the PSL mice compared with that in the sham mice (Figure 4(a)). This increased expression was significantly inhibited by yokukansan extract (1 g/kg) and a mixture of Glycyrrhizae Radix (100 mg/kg) and Atractylodis Lanceae Rhizoma (200 mg/kg) extracts in the PSL mice (Figures 4(b) and 4(c)). In addition, repetitive administration of Atractylodis Lanceae Rhizoma extract (200 mg/kg), but not Glycyrrhizae Radix extract (100 mg/kg), also decreased IL-6 mRNA expression in the spinal cord of the PSL mice (Figures 4(d) and 4(e)).

### 3.4. Expression and Distribution of IL-6 in the Dorsal Horn of the Spinal Cord in PSL Mice

**Figure 5:**
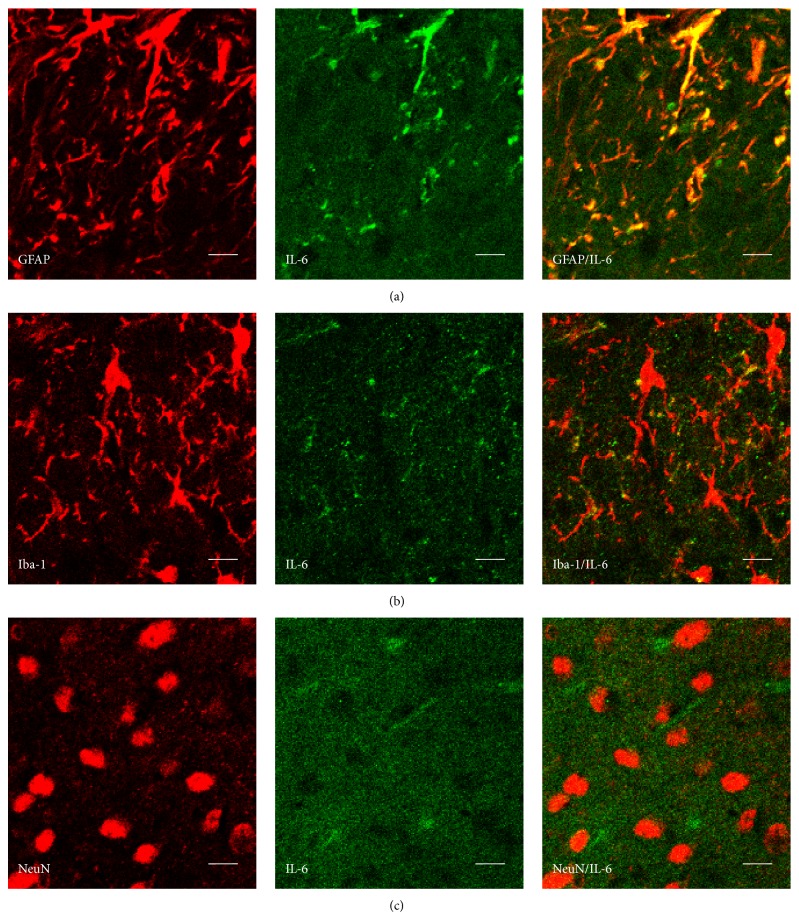
Typical example of the distribution of interleukin- (IL-) 6 in the dorsal horn of the lumbar spinal cord in mice with mechanical allodynia induced by partial sciatic nerve ligation (PSL). The sections of the lumbar spinal cord are double-immunostained with IL-6 (green) and glial fibrillary acidic protein (GFAP) ((a) red), ionized calcium-binding adaptor molecule 1 (Iba-1) ((b) red), or NeuN ((c) red). Scale bars = 10 *μ*m.

Immunohistochemical staining in the dorsal horn of the spinal cord in PSL mice was performed 11 days after surgery. IL-6 was primarily expressed in GFAP-immunoreactive astrocytes and Iba-I-immunoreactive microglia (Figures 5(a) and 5(b)). NeuN-immunoreactive neurons did not show IL-6 expression (Figure 5(c)).

## 4. Discussion

The present study showed that the repetitive oral administration of yokukansan extract alleviated mechanical allodynia in PSL mice. In our preliminary experiment, repetitive keishikajutsubuto administration also exerted therapeutic effects on mechanical allodynia (data not shown). Therefore, we also examined whether extracts of the herbal ingredients (Atractylodis Lanceae Rhizoma and Glycyrrhizae Radix) in these Kampo medicines inhibited mechanical allodynia in PSL mice and found that the repetitive administration of Atractylodis Lanceae Rhizoma extract, but not Glycyrrhizae Radix extract, attenuated mechanical allodynia in PSL mice, suggesting that Atractylodis Lanceae Rhizoma is involved in the antiallodynic effects of yokukansan.

Although the extract of Glycyrrhizae Radix did not show antiallodynic effects in the PSL mice in this study, a mixture of Atractylodis Lanceae Rhizoma and Glycyrrhizae Radix extracts was more effective than Atractylodis Lanceae Rhizoma extract alone. Glycyrrhizae Radix extract reportedly enhances the antiallodynic effects of Paeoniae Radix [19]. In addition, itexhibits synergistic antioxidant activities with the other herbal medicines [20]. Taken together, Glycyrrhizae Radix may play an important role in the enhancement of the therapeutic effects of the other constituent herbal medicines.

Spinal cord IL-6 contributes to mechanical allodynia in PSL mice [10]. Therefore, we investigated whether the regulation of IL-6 was involved in the antiallodynic action of yokukansan extract in PSL mice. Yokukansan extract did not attenuate intrathecal IL-6-induced mechanical allodynia. Thus, it is suggested that yokukansan extract does not affect IL-6-induced action, at least mechanical allodynia. As another possibility, we examined whether the extracts of yokukansan and the constituent herbal medicines regulated the expression of IL-6 mRNA. In this study, the increased expression of IL-6 mRNA in the spinal cord of the PSL mice was inhibited by the repetitive administration of yokukansan extract, a mixture of Atractylodis Lanceae Rhizoma and Glycyrrhizae Radix extracts, and Atractylodis Lanceae Rhizoma extract alone, but not by the administration of Glycyrrhizae Radix extract alone. The detailed mechanisms underlying the regulation of IL-6 mRNA expression remain unknown. However, it has been reported in an in vitro study that *β*-eudesmol, a component of Atractylodis Lanceae Rhizoma, inhibits phorbol 12-myristate 13-acetate-induced IL-6 expression through the suppression of p38 mitogen-activated protein kinase (p38 MAPK) and nuclear factor kappa B (NF*κ*B) activation [21]. Therefore, it is suggested that the regulation of p38 MAPK and NF*κ*B activation by the components (e.g., *β*-eudesmol) of Atractylodis Lanceae Rhizoma, which is one of the constituent herbal medicines of yokukansan, may be involved in the inhibition of IL-6 expression.

Microglia and astrocytes in the spinal cord play an important role in neuropathic pain [10, 22–24]. In this study, IL-6 immunoreactivity was detected in GFAP-immunoreactive astrocytes and Iba-I-immunoreactive microglia, but not in NeuN-immunoreactive neurons, in the dorsal horn of the lumbar spinal cord of the PSL mice. In particular, the immunoreactivity of IL-6 in astrocytes was higher than that in microglia. These findings suggest that the components (e.g., *β*-eudesmol) of Atractylodis Lanceae Rhizoma, which is one of the constituent herbal medicines in yokukansan, may suppress IL-6 expression in the astrocytes and/or microglia of PSL mice.

## 5. Conclusions

Yokukansan and its herbal ingredient Atractylodis Lanceae Rhizoma exhibited therapeutic effects in mouse models of PSL-induced mechanical allodynia. The inhibition of IL-6 expression in astrocytes and/or microglia in the spinal cord may be involved in the antiallodynic effects of yokukansan, while the components (e.g., *β*-eudesmol) of Atractylodis Lanceae Rhizoma may be involved in the regulation of IL-6 expression.

## Figures and Tables

**Table 1:** Herbal medicines composing yokukansan.

	Herbal medicine	(g)
Atractylodis Lanceae Rhizoma	*Atractylodes lancea* De Candolle	4.0
Hoelen	*Poria cocos* Wolf	4.0
Cnidii Rhizoma	*Cnidium officinale* Makino	3.0
Uncariae Uncis Cum Ramulus	*Uncaria rhynchophylla* Miquel	3.0
Angelicae Radix	*Angelica acutiloba* Kitagawa	3.0
Bupleuri Radix	*Bupleurum falcatum* Linn$\overset{´}{\text{e}}$	2.0
Glycyrrhizae Radix	*Glycyrrhiza uralensis* Fisher	1.5
